# Age-related differences in the cloacal microbiota of a wild bird species

**DOI:** 10.1186/1472-6785-13-11

**Published:** 2013-03-25

**Authors:** Wouter FD van Dongen, Joël White, Hanja B Brandl, Yoshan Moodley, Thomas Merkling, Sarah Leclaire, Pierrick Blanchard, Étienne Danchin, Scott A Hatch, Richard H Wagner

**Affiliations:** 1 Konrad Lorenz Institute of Ethology, Department of Integrative Biology and Evolution, University of Veterinary Medicine Vienna, Savoyenstrasse 1a, Vienna, 1160, Austria; 2 CNRS-UPS-ENFA; Laboratoire Évolution & Diversité Biologique (EDB), UMR 5174, 118 Route de Narbonne, Toulouse, F-31062, France; 3 US Geological Survey, Alaska Science Center, 4210 University Drive, Anchorage, AK, 99508, USA

**Keywords:** Age-differences, Automated ribosomal intergenic spacer analysis, Bacteria, Black-legged kittiwakes, Cloaca, Gastrointestinal tract

## Abstract

**Background:**

Gastrointestinal bacteria play a central role in the health of animals. The bacteria that individuals acquire as they age may therefore have profound consequences for their future fitness. However, changes in microbial community structure with host age remain poorly understood. We characterised the cloacal bacteria assemblages of chicks and adults in a natural population of black-legged kittiwakes (*Rissa tridactyla*), using molecular methods.

**Results:**

We show that the kittiwake cloaca hosts a diverse assemblage of bacteria. A greater number of total bacterial OTUs (operational taxonomic units) were identified in chicks than adults, and chicks appeared to host a greater number of OTUs that were only isolated from single individuals. In contrast, the number of bacteria identified per individual was higher in adults than chicks, while older chicks hosted more OTUs than younger chicks. Finally, chicks and adults shared only seven OTUs, resulting in pronounced differences in microbial assemblages. This result is surprising given that adults regurgitate food to chicks and share the same nesting environment.

**Conclusions:**

Our findings suggest that chick gastrointestinal tracts are colonised by many transient species and that bacterial assemblages gradually transition to a more stable adult state. Phenotypic differences between chicks and adults may lead to these strong differences in bacterial communities. These data provide the framework for future studies targeting the causes and consequences of variation in bacterial assemblages in wild birds.

## Background

In animals, bacterial communities occur both externally (e.g. skin, feather, scales) and internally (e.g. gastrointestinal and reproductive tracts) of their hosts [1]. Although many gastrointestinal bacteria are host commensal, others are adaptive by being central in fundamental physiological processes [2-4], or have pathogenic effects [5]. Given the potentially profound impact of the gastrointestinal microbiota on host condition, the study of bacterial assemblages within animals is of high ecological and evolutionary importance [6].

One of the central questions in the study of microbial communities in animals is how they are acquired and how the structure of communities varies with age. Young animals either passively or actively acquire microbiota from the environment after birth or hatching [6-8]. The gaxstrointestinal tract of young individuals is typically characterised by a high number of transient microbial species before eventually transitioning to a relatively stable state as adults [e.g. [9]. However, it remains unclear how microbial communities change throughout the lifetime of their hosts and how these fluctuations affect host health, behaviour and fitness [6]. Temporal variation in microbial composition may have important consequences for factors such as nutrition e.g. [8,10], mating preferences e.g. [11] and susceptibility to diseases e.g. [12]. It is therefore necessary to characterise bacterial communities at different life history stages of animal hosts. Scant information exists, however, and much past research has focussed on humans or other mammal species [7,8].

Birds comprise a useful model for studying the causes and consequences of variation in bacterial assemblages. For example, the contrasting life histories of birds and mammals may result in differences in the mechanisms of microbial colonisation. Mammals, for example, acquire important maternal microbes during the birth process [9], whereas birds are more likely to acquire microbes after hatching from other sources, such as the nesting environment or food [7]. Second, many bird species regurgitate food to their young, thus permitting a mode of vertical microbial transmission that most mammals lack. Last, birds possess a cloaca, which serves the dual functions of excretion and gamete transfer. Gastrointestinal bacteria may thus be transferred during copulation [13], providing the potential for pathogens [14,15] or beneficial strains to be sexually transmitted [16].

The few avian studies on the effect of age on gastrointestinal assemblages have mostly been conducted on domestic birds e.g. [17-20]. However, the artificial rearing conditions in which captive animals are raised make it difficult to extrapolate knowledge of bacterial assemblages in captive animals to their wild counterparts [21,22]. Moreover, the few studies in wild birds typically examine only a few target bacterial species and do not use community-level approaches to test for host age differences in bacterial assemblages e.g. [23-26], but see [10]. Very little is therefore known about how the normal gastrointestinal bacterial communities change between young and adult wild birds. This information forms an important basis for understanding bacterial acquisition and transmission mechanisms, the prevalence of enteropathogens and the consequences of particular communities of bacteria for host health.

Here, we characterise the bacterial assemblages of both adults and chicks in a wild population of black-legged kittiwakes (*Rissa tridactyla*), a pelagic gull with a wide distribution. We focus our analyses on cloacal assemblages because they are known to approximate assemblages deeper within the gastrointestinal tract [27] while requiring relatively non-invasive sampling methods. We have previously used a molecular technique [ARISA - Automated Ribosomal Intergenic Spacer Analysis: [28] to explore variation in cloacal bacteria assemblages in kittiwakes [13]. Although ARISA allows the rapid assessment of bacterial assemblage diversity, it does not permit species identification. We have therefore further developed a comprehensive ribosomal RNA (rRNA) clone library to identify actual bacterial species. Combined with ARISA, this allows the rapid assessment of actual species in bacterial assemblages, thus providing a framework for studying the causes and consequences of microbial assemblage variation in kittiwakes. We then use a community-based approach to test whether age predicts bacterial assemblage composition in kittiwakes and which bacterial lineages differ between chicks and adults.

## Methods

Cloacal bacterial assemblages were sampled from 22 adult and 21 black-legged kittiwake chicks captured at nests in an abandoned U.S. Air Force radar tower on Middleton Island (59° 26’ N, 146° 20’ W) in the Gulf of Alaska [13]. As an aim of our study was to taxonomically classify ARISA peaks (see below), it was necessary to obtain a comprehensive sample of the most common bacterial species present in chicks and adults. We therefore only sampled unrelated individuals (to minimise the overlap in bacterial assemblages between two individuals due to, for example, a shared nesting environment). In addition, chicks of a wide range of ages were sampled (range = 5–30 days, mean = 16.0 ± 8.3 SD days) as bacterial assemblages were hypothesised to change rapidly with age. Sampling occurred between May and July 2009. Cloacal sampling and DNA extraction protocols are outlined in detail in White et al. 2010 [13]. We also regularly collected control samples during field work by pipetting 1 ml of saline solution into a sterile vial from the saline stock, which ensured that all bacteria identified were of cloacal origin and not due to contamination of the stock solution or of reagents used for DNA extraction.

### Automated ribosomal intergenic spacer analysis

We first characterised cloacal bacterial assemblages by performing PCR-ARISA, which exploits the extreme interspecies variability in the length of the intergenic spacer (IGS) lying between the conserved 16S and 23S rRNA genes in the bacterial ribosomal operon. ARISA involves the amplification of DNA extracted from the bacterial assemblage of interest using a fluorescently-labelled primer and subsequent high-resolution electrophoresis in an automated system. Assemblages are therefore characterised by a series of electrophoretic peaks that vary according to the length of the amplified IGS fragment of each bacterial species. Given that unrelated species may share the same IGS fragment length, the number of species represented by each ARISA peak remains uncertain. This method has been optimised for the characterisation of cloacal bacterial assemblages in kittiwakes [13]. Briefly, the 16S-23S rRNA intergenic spacer was amplified using the FAM (6-carboxyfluorescein)-labeled primer S-D-Bact-1522-b-S-20 (5’-[6FAM] TGCGGCTGGATCCCCTCCTT-3’) and the unlabelled L-D-Bact-132-a- A-18 (5’-CCGGGTTTCCCCATTCGG-3’). In addition to amplifying the highly variable IGS region, the primers also amplify a 131 bp fragment of the 23S rRNA gene, which we used for subsequent phylogenetic analyses (see below).

Fragment amplification was performed using PCR in 20 μl reactions containing 0.2 mM dNTPs, 1.5 mM MgCl_2_, 0.25 μM of each primer, and 1 unit of GoTaq DNA polymerase with 1× buffer (Promega). Due to the coextraction of host and environmental DNA, it was not possible to accurately measure the concentration of the extracted DNA. Therefore, a standard of 4 μl of eluted DNA extract was used per reaction. PCR was conducted using the following program: 94°C for 10 minutes, followed by 30 cycles of 94°C for 1 minute, 55°C for 1 minute and 72°C for 1 minute, and a final elongation step at 72°C for 10 minutes. The successful amplification of the samples was confirmed on a 2% agarose gel and all PCR products were then diluted by a ratio of 1 in 30. One microlitre of the diluted product was then added to 8.7 μl Hidi Formamide (Applied Biosystems) and 0.3 μl of in-house designed ROX size standard (size standard range = 105 – 1007 bp). The mixture was then run on an ABI PRISM 3130×l automated sequencer (Applied Biosystems). Traces were viewed on GeneMapper software 4.0 (Applied Biosystems).

### IGS-23S rRNA library construction and analysis

We created an IGS-23S rRNA clone library of our samples in order to taxonomically classify ARISA peaks. We defined three hierarchical levels of units in our analyses: ARISA peaks, clones and OTUs (operational taxonomic units). As stated above, an ARISA peak is defined as one electrophoretic peak in an ARISA profile. Cloned fragments in our IGS-23S rRNA library were referred to as clones. During each cloning event we typically isolated multiple copies of the same sequence. Each unique clone (i.e. those with a unique sequence) was assumed to correspond to one unique ARISA peak. Finally, when multiple unique clones shared the same 23S rRNA sequence and differed from each other only within the IGS, we assumed that they belonged to the same OTU. This approach in OTU classification is conservative but justified given that many bacterial species have multiple operons in their genomes that result in multiple ARISA peaks per OTU [29].

In developing our rRNA library, IGS-23S fragments of our bacterial samples were amplified as outlined above, except that both primers were unlabelled. Amplified products were then cloned using a TOPO TA cloning kit (Invitrogen) as per the manufacturer’s protocol. We then picked between 8 and 96 transformed colonies for each cloacal sample and amplified the inserts using colony PCR in 25 μl reactions containing 2.5 mM MgCl_2_, 0.1 mM dNTPs, 0.2 μM M13 forward and reverse primer, and 2.5 units of Firepol polymerase and 1× buffer (Solis BioDyne). Reactions were run at 94°C for 5 minutes, followed by 35 cycles of 94°C for 30 s, 55°C for 30 s and 72°C for 30 s, and a final extension at 72°C for 10 min. The successful amplification of the samples was confirmed on a 2% agarose gel and primers and excess dNTPs were removed from the amplified products by digestion with exonuclease-shrimp and alkaline phosphatase (Fermentas Life Sciences). Cloned PCR products were amplified in both directions using Big Dye chemistry (Applied Biosystems) and sequenced on an ABI PRISM 3130×l automated sequencer (Applied Biosystems). Sequence editing and alignment was conducted using CLC DNA Workbench 6.1 (CLC bio). Rarefaction analyses were conducted using Analytic Rarefaction 1.3 (available at http://strata.uga.edu/software) to ascertain the comprehensiveness of our cloning effort.

### Comparison of ARISA peak size and clone fragment lengths

One aim of our study was to identify the bacterial OTUs present in each cloacal sample of chicks and adults based entirely on the ARISA output. We therefore compared the length of cloned sequences with the peaks present in the ARISA profiles. We assigned each clone for each individual to an ARISA peak, taking into account the presence or absence of the ARISA peak and clone sequence in the other cloned samples for that age class. Any peak to which we did not assign a sequence was assumed to be an artefact and was therefore eliminated from subsequent analyses.

### Bacterial taxonomy

A Basic Local Alignment Search Tool search BLAST: [30] was implemented on the 23S rRNA sequence of cloned fragments to screen for published sequences that closely match our cloned sequences. The maximum BLAST score (typically > 200) and Expect-value (typically < 1 × 10^-50^) were used as indicators of the strength of sequence similarity.

As BLAST provides information on similarity between two sequences but not on relatedness among sequences, we employed phylogenetic analyses to infer the taxonomic classification of the clones. We included all unique clones as well as two to four similar sequences for each clone from known species identified by BLAST. Both maximum-likelihood and Bayesian methods were used to ascertain the phylogenetic position of each unidentified species relative to known bacterial species. Given the immense diversity among bacteria, we conducted analyses separately for each phylum identified (Firmicutes, Actinobacteria and Proteobacteria). It was necessary to further separate the Proteobacteria into classes (Alphaproteobacteria, Betaproteobacteria and Gammaproteobacteria). Maximum-likelihood trees were constructed in Treefinder [31] using the nucleotide substitution model suggested by the Bayesian information criterion. Bootstrap support was estimated after 1000 replicates. Bayesian phylogenies were constructed in MrBayes 3.1.2 [32] using the same substitution model as suggested by Treefinder. Posterior probabilities were estimated after 10 million generations, sampling every 1000 generations.

The most derived phylogenetic position for each clone was then estimated based on a combination of the BLAST result and the phylogenetic trees. For example, when both BLAST and the phylogeny agreed that a particular clone belonged to a specific genus, we classified the clone to that genus. However, if there was disagreement between BLAST and the phylogenetic tree in the phylogenetic position of the clone, we classified the clone to a higher taxonomic level (e.g. class or family), with an emphasis on the position of the clone within the tree. Full sequences for each OTU have been deposited in GenBank under Accession numbers KC462595 - KC462734.

### Bacterial assemblage comparisons

To characterise differences in the bacterial assemblages between chicks and adults, we implemented Unifrac [33] to compare overall bacterial assemblage composition using phylogenetic information. All assemblage analyses were conducted on the ARISA data and without abundance weights. Using the cloning data may produce misleading results due to stochastic variation in the identity of sequences cloned for each individual.

A 23S rRNA phylogenetic tree containing all sequences found in chicks and adults was used for the analyses. We then implemented Unifrac to test 1) whether bacterial assemblages cluster by age (i.e. adults and chicks) using both the environmental clustering analysis (setting ‘number of sequences to keep’ to five and ‘number of permutations’ to 100) and principal coordinates analysis, 2) whether any clustering is statistically significant using both the P test (which does not use phylogenetic information; 100 permutations) and the Unifrac test (which tests whether more evolutionary history of bacterial assemblages is shared within age classes than between classes; 100 permutations) and 3) which bacterial lineages are responsible for differences in assemblages between the age classes, via the lineage-specific analysis (setting ‘minimum descendants’ to four).

A greater quantity of bacterial DNA could be extracted from adults than chicks (data not shown), probably due to the larger volume of saline solution that could be used to flush adult cloacae. Cloning of bacterial DNA fragments therefore tended to be more successful for adults than chicks and twice as many clones were sequenced per individual for adults compared to chicks (number of clones sequenced: adults – 47.0±28.0 clones, chicks – 20.2±6.7 clones, Mann Whitney U = 434.0, P<0.001). This age-related discrepancy could lead to biases in downstream analyses in, for example, our estimates of age differences in the number of species hosted per individual. This, however, does not appear to be the case. First, the proportion of ARISA peaks that were discarded because they could not be assigned OTUs did not differ between adults and chicks (proportion of peaks discarded per individual: adults – 0.63±0.13, chicks – 0.66±0.14, F_1,41_=0.534, P=0.469), although the absolute number discarded was higher in adults (number of peaks discarded per individual: adults – 12.7±5.9 peaks, chicks – 7.6±1.6 peaks, Mann Whitney U = 101.0, P=0.001) probably because higher quantities of bacterial DNA in adults produced more noise in the ARISA profiles. Second, cloning effort did not differ between chicks of different ages, both when correlating chick age with number of clones sequenced per individual (r = 0.273, F_1,19_=1.528, P=0.231) or in a univariate test grouping chicks into young (5–10 days old) and old individuals (20–30 days old; number of clones sequenced per individual – young chicks: 18.5±4.5 clones, old chicks: 22.4±8.3 clones, F_1,19_=1.966, P=0.177). Last, our Unifrac analyses are unaffected by these potential biases as we kept a minimum of five sequences for the clustering analyses and ran 100 permutations on the data. All non-genetic statistical analyses were conducting using SPSS 17.0 (SPSS, Chicago, Illinois, USA). All data are presented as mean ± standard deviation.

## Results

### Comparison of ARISA peak size and clone fragment lengths

A mean of 33.9 ± 24.4 clones per individual were sequenced (range = 8 – 120 clones per individual). Clones ranged in size from 304 bp to 1090 bp (Table 1). In total, we identified 142 unique clones, of which 99 were successfully matched to ARISA peaks. In the remaining 43 cases, we were unable to match peaks to clones because the clone was rare, resulting in low confidence of assignment. Due to the relatively high proportion of OTUs for which we could not assign ARISA peaks, the analyses presented below (e.g. differences in bacterial assemblages between adults and chicks) apply only to the most common OTUs found in chicks and adults.

**Table 1:** Identity of OTUs isolated from cloacae of black-legged kittiwakes

**OTU**	**ARISA peak**	**Clone**	**Difference**	**BLAST match**	**BLAST score**	**Most derived phylogenetic position**	**Phylum**	**Found in**
1	304	308	+4	*Peptoniphilus asaccharolyticus*	152	Genus: *Peptoniphilus*	Firmicutes	Ad
1	305	309	+4	*Peptoniphilus asaccharolyticus*	152	Genus: *Peptoniphilus*	Firmicutes	Ad
1	396	399	+3	*Peptoniphilus asaccharolyticus*	152	Genus: *Peptoniphilus*	Firmicutes	Ad
1	397	400	+3	*Peptoniphilus asaccharolyticus*	152	Genus: *Peptoniphilus*	Firmicutes	Ad
1	398	401	+3	*Peptoniphilus asaccharolyticus*	152	Genus: *Peptoniphilus*	Firmicutes	Ad
2	313	315	+2	*Enterococcus pseudoavium*	228	Genus: *Enterococcus*	Firmicutes	Ch
3	331	331	0	*Enterococcus faecalis*	211	Order: Lactobacillales	Firmicutes	Ad, Ch
3	432	433	+1	*Enterococcus faecalis*	211	Order: Lactobacillales	Firmicutes	Ad, Ch
4	340	341	+1	*Enterococcus malodoratus*	239	Genus: *Enterococcus*	Firmicutes	Ch
4	341	342	+1	*Enterococcus malodoratus*	239	Genus: *Enterococcus*	Firmicutes	Ch
4	433	434	+1	*Enterococcus malodoratus*	239	Genus: *Enterococcus*	Firmicutes	Ch
5	345	342	−3	*Corynebacterium propinquum*	239	Genus: *Corynebacterium*	Actinobacteria	Ad
5	346	343	−3	*Corynebacterium propinquum*	239	Genus: *Corynebacterium*	Actinobacteria	Ad
5	347	344	−3	*Corynebacterium propinquum*	239	Genus: *Corynebacterium*	Actinobacteria	Ad
5	348	345	−3	*Corynebacterium propinquum*	239	Genus: *Corynebacterium*	Actinobacteria	Ad
6		342		*Enterococcus malodoratus*	206	Order: Lactobacillales	Firmicutes	Ch
7	348	349	+1	*Arthromitus* sp.	233	Genus: *Arthromitus*	Firmicutes	Ad, Ch
7	418	419	+1	*Arthromitus* sp.	233	Genus: *Arthromitus*	Firmicutes	Ad, Ch
8		349		*Lactobacillus salivarius*	206	Genus: *Lactobacillus*	Firmicutes	Ch
9	349	350	+1	*Lactobacillus animalis*	213	Genus: *Lactobacillus*	Firmicutes	Ad, Ch
9	440	443	+3	*Lactobacillus animalis*	213	Genus: *Lactobacillus*	Firmicutes	Ad, Ch
9	441	444	+3	*Lactobacillus animalis*	213	Genus: *Lactobacillus*	Firmicutes	Ad, Ch
9	519	520	+1	*Lactobacillus animalis*	213	Genus: *Lactobacillus*	Firmicutes	Ad, Ch
9	520	521	+1	*Lactobacillus animalis*	213	Genus: *Lactobacillus*	Firmicutes	Ad, Ch
10	352	353	+1	*Aerococcus urinae*	222	Order: Lactobacillales	Firmicutes	Ad
11	357	354	−3	*Corynebacterium aurimucosum*	196	Genus: *Corynebacterium*	Actinobacteria	Ad
12		357		*Atopobium parvulum*	204	Order: Clostridiales	Firmicutes	Ad
13		357		*Carnobacterium mobile*	237	Genus: *Carnobacterium*	Firmicutes	Ch
13		463		*Carnobacterium mobile*	237	Genus: *Carnobacterium*	Firmicutes	Ch
14		357		*Lactobacillus crispatus*	237	Genus: *Lactobacillus*	Firmicutes	Ad
14	603	607	+4	*Lactobacillus crispatus*	237	Genus: *Lactobacillus*	Firmicutes	Ad
15		361		*Bacillus megaterium*	215	Genus: *Bacillus*	Firmicutes	Ch
16	365	361	−4	*Mycobacterium sp.*	161	Suborder: Corynebacterineae	Actinobacteria	Ad
16	366	362	−4	*Mycobacterium sp.*	161	Suborder: Corynebacterineae	Actinobacteria	Ad
16	367	363	−4	*Mycobacterium sp.*	161	Suborder: Corynebacterineae	Actinobacteria	Ad
16	368	364	−4	*Mycobacterium sp.*	161	Suborder: Corynebacterineae	Actinobacteria	Ad
16	369	365	−4	*Mycobacterium sp.*	161	Suborder: Corynebacterineae	Actinobacteria	Ad
16	371	367	−4	*Mycobacterium sp.*	161	Suborder: Corynebacterineae	Actinobacteria	Ad
17		363		*Oceanobacillus iheyensis*	187	Class: Bacilli	Firmicutes	Ch
18	380	375	−5	*Arcanobacterium abortisuis*	174	Family: Actinomycetaceae	Actinobacteria	Ad
19		377		*Clostridium difficile*	217	Genus: *Clostridium*	Firmicutes	Ch
20	376	378	+2	*Enterococcus faecalis*	239	Genus: *Enterococcus*	Firmicutes	Ch
20	478	480	+2	*Enterococcus faecalis*	239	Genus: *Enterococcus*	Firmicutes	Ch
21	392	388	−4	*Corynebacterium aurimucosum*	182	Genus: *Corynebacterium*	Actinobacteria	Ad
22	388	390	+2	*Pediococcus inopinatus*	174	Order: Lactobacillales	Firmicutes	Ch
23	392	395	+3	*Clostridium piliforme*	219	Genus: *Clostridium*	Firmicutes	Ch
23	393	396	+3	*Clostridium piliforme*	219	Genus: *Clostridium*	Firmicutes	Ch
24		398		*Acidimicrobineae* sp.	180	Phylum: Actinobacteria	Actinobacteria	Ad
25		400		*Clostridium piliforme*	209	Genus: *Clostridium*	Firmicutes	Ad
25	753	756	+3	*Clostridium piliforme*	209	Genus: *Clostridium*	Firmicutes	Ad
26	398	401	+3	*Staphylococcus vitulinus*	243	Genus: *Staphylococcus*	Firmicutes	Ch
26	399	402	+3	*Staphylococcus vitulinus*	243	Genus: *Staphylococcus*	Firmicutes	Ch
26	406	407	+1	*Staphylococcus vitulinus*	243	Genus: *Staphylococcus*	Firmicutes	Ch
26	430	431	+1	*Staphylococcus vitulinus*	243	Genus: *Staphylococcus*	Firmicutes	Ch
26	431	432	+1	*Staphylococcus vitulinus*	243	Genus: *Staphylococcus*	Firmicutes	Ch
26	489	492	+2	*Staphylococcus vitulinus*	243	Genus: *Staphylococcus*	Firmicutes	Ch
26	520	520	0	*Staphylococcus vitulinus*	243	Genus: *Staphylococcus*	Firmicutes	Ch
26	521	521	0	*Staphylococcus vitulinus*	243	Genus: *Staphylococcus*	Firmicutes	Ch
26		621		*Staphylococcus vitulinus*	243	Genus: *Staphylococcus*	Firmicutes	Ch
27	421	422	+1	*Bacillus* sp.	213	Genus: *Bacillus*	Firmicutes	Ch
27	422	423	+1	*Bacillus* sp.	213	Genus: *Bacillus*	Firmicutes	Ch
27	570	568	+2	*Bacillus* sp.	213	Genus: *Bacillus*	Firmicutes	Ch
27	589	590	+1	*Bacillus* sp.	213	Genus: *Bacillus*	Firmicutes	Ch
28		434		*Streptococcus parauberis*	231	Genus: *Streptococcus*	Firmicutes	Ch
29		438		*Acidimicrobiaceae* sp.	189	Phylum: Actinobacteria	Actinobacteria	Ch
30	437	439	+2	*Staphylococcus saprophyticus*	239	Genus: *Staphylococcus*	Firmicutes	Ch
30	438	440	+2	*Staphylococcus saprophyticus*	239	Genus: *Staphylococcus*	Firmicutes	Ch
31	446	441	−5	*Corynebacterium propinquum*	182	Genus: *Corynebacterium*	Actinobacteria	Ad
32	443	445	+3	*Bacillus globisporus*	206	Genus: *Bacillus*	Firmicutes	Ch
33		446		*Bacillus cereus*	213	Genus: *Bacillus*	Firmicutes	Ch
34	452	448	−4	*Corynebacterium propinquum*	172	Genus: *Corynebacterium*	Actinobacteria	Ad
34	458	454	−4	*Corynebacterium propinquum*	172	Genus: *Corynebacterium*	Actinobacteria	Ad
34	459	455	−4	*Corynebacterium propinquum*	172	Genus: *Corynebacterium*	Actinobacteria	Ad
34	460	456	−4	*Corynebacterium propinquum*	172	Genus: *Corynebacterium*	Actinobacteria	Ad
34	461	457	−4	*Corynebacterium propinquum*	172	Genus: *Corynebacterium*	Actinobacteria	Ad
35	453	454	+1	*Corynebacterium amycolatum*	200	Genus: *Corynebacterium*	Actinobacteria	Ad
35	454	455	+1	*Corynebacterium amycolatum*	200	Genus: *Corynebacterium*	Actinobacteria	Ad
36	480	479	−1	*Rhodococcus erythropolis*	207	Genus: *Rhodococcus*	Actinobacteria	Ad
36	490	490	0	*Rhodococcus erythropolis*	207	Genus: *Rhodococcus*	Actinobacteria	Ad
36	493	492	−1	*Rhodococcus erythropolis*	207	Genus: *Rhodococcus*	Actinobacteria	Ad
37	502	505	+3	*Escherichia coli*	213	Genus: *Escherichia*	Proteobacteria	Ad, Ch
37	504	507	+3	*Escherichia coli*	213	Genus: *Escherichia*	Proteobacteria	Ad, Ch
37	506	509	+3	*Escherichia coli*	213	Genus: *Escherichia*	Proteobacteria	Ad, Ch
37	596	599	+3	*Escherichia coli*	213	Genus: *Escherichia*	Proteobacteria	Ad, Ch
37	598	601	+3	*Escherichia coli*	213	Genus: *Escherichia*	Proteobacteria	Ad, Ch
38		508		*Bacillus globisporus*	198	Genus: *Bacillus*	Firmicutes	Ch
39		509		*Nocardia seriolae*	174	Order: Actinomycetales	Actinobacteria	Ch
39		514		*Nocardia seriolae*	174	Order: Actinomycetales	Actinobacteria	Ch
40	520	522	+2	*Corynebacterineae* sp.	207	Order: Actinomycetales	Actinobacteria	Ad
41	527	526	−1	*Microbacterium testaceum*	198	Genus: *Microbacterium*	Actinobacteria	Ch
42	542	536	−2	*Rothia mucilaginosa*	174	Genus: *Rothia*	Actinobacteria	Ad, Ch
42	544	538	−2	*Rothia mucilaginosa*	174	Genus: *Rothia*	Actinobacteria	Ad, Ch
43	544	545	+1	*Staphylococcus aureus*	207	Genus: *Staphylococcus*	Firmicutes	Ad
44		546		*Nocardia seriolae*	130	Order: Actinomycetales	Actinobacteria	Ch
45		554		*Lactobacillus mucosae*	209	Genus: *Lactobacillus*	Firmicutes	Ad
46	572	577	+5	*Clostridium tyrobutyricum*	90	Order: Clostridiales	Firmicutes	Ad
47		580		*Streptococcus pyogenes*	198	Genus: *Streptococcus*	Firmicutes	Ad, Ch
47	707	712	+5	*Streptococcus pyogenes*	198	Genus: *Streptococcus*	Firmicutes	Ad, Ch
47	713	718	+5	*Streptococcus pyogenes*	198	Genus: *Streptococcus*	Firmicutes	Ad, Ch
47	716	721	+5	*Streptococcus pyogenes*	198	Genus: *Streptococcus*	Firmicutes	Ad, Ch
47	785	788	+3	*Streptococcus pyogenes*	198	Genus: *Streptococcus*	Firmicutes	Ad, Ch
48	592	591	−1	*Micrococcus luteus*	213	Genus: *Micrococcus*	Actinobacteria	Ch
49	611	609	−2	*Corynebacterium efficiens*	169	Genus: *Corynebacterium*	Actinobacteria	Ch
50	611	614	+3	*Streptococcus dysgalactiae*	213	Genus: *Streptococcus*	Firmicutes	Ad
51		615		*Jonesia denitrificans*	182	Suborder: Micrococcineae	Actinobacteria	Ch
51		620		*Jonesia denitrificans*	182	Suborder: Micrococcineae	Actinobacteria	Ch
51		621		*Jonesia denitrificans*	182	Suborder: Micrococcineae	Actinobacteria	Ch
51		622		*Jonesia denitrificans*	182	Suborder: Micrococcineae	Actinobacteria	Ch
52	622	625	+3	Sinobacteraceae sp.	211	Order: Xanthomonadales	Proteobacteria	Ad
53		626		*Agrococcus* sp.	207	Family: Microbacteriaceae	Actinobacteria	Ch
54		634		*Clavibacter michiganensis*	185	Family: Microbacteriaceae	Actinobacteria	Ch
55		670		*Yersinia enterocolitica*	215	Genus: *Yersinia*	Proteobacteria	Ad
56		671		*Herbaspirillum* sp.	217	Genus: *Herbaspirillum*	Proteobacteria	Ad
57		674		*Arthrobacter arilaitensis*	198	Family: Micrococcaceae	Actinobacteria	Ch
58		692		*Azoarcus* sp.	195	Family: Rhodocyclaceae	Proteobacteria	Ch
59		713		*Agromyces* sp.	185	Family: Microbacteriaceae	Actinobacteria	Ch
60		723		*Micrococcus luteus*	176	Family: Micrococcaceae	Actinobacteria	Ch
61		740		*Arthrobacter arilaitensis*	182	Family: Micrococcaceae	Actinobacteria	Ch
62	794	796	+2	*Phenylobacterium zucineum*	165	Family: Caulobacteraceae	Proteobacteria	Ad
63	973	974	+1	*Bradyrhizobium elkanii*	209	Genus: *Bradyrhizobium*	Proteobacteria	Ad, Ch
63		1047		*Bradyrhizobium elkanii*	209	Genus: *Bradyrhizobium*	Proteobacteria	Ad, Ch
63		1048		*Bradyrhizobium elkanii*	209	Genus: *Bradyrhizobium*	Proteobacteria	Ad, Ch
64		1090		Bradyrhizobiaceae sp.	198	Genus: *Bradyrhizobium*	Proteobacteria	Ch

For each OTU, the following variables are displayed: OTU identification number, ARISA peak size, clone fragment length, the difference between the latter two variables (i.e. clone length minus ARISA peak size), the species identity of the most similar sequence in GenBank as determined by the BLAST algorithm, the maximum BLAST score, the most derived phylogenetic position of the OTU and the phylum to which the OTU belongs. In some cases, we could not determine which ARISA peak represented a particular clone (indicated by a blank space in the “ARISA peak” column).

The 142 unique clones corresponded to 76 OTUs. This discrepancy was due to indels causing variation in clone lengths for several OTUs. For example, although some OTUs were always consistent in clone length (e.g. OTU 2, Table 1), others were more variable (e.g. OTU 5 varied between 342 and 345 bp in length). In most cases, one ARISA peak corresponded to a single OTU. However, in 16 cases, one ARISA peak was assigned to two distantly-related OTUs. For example, the 348 bp peak corresponded to species within the genera *Corynebacterium* and *Athromitus*, while the 544 bp peak corresponded to species of *Rothia* and *Staphylococcus.* The mean number of ARISA peaks per OTU was 1.5 ± 1.5 (range 1–9). Of the 76 OTUs identified, 64 were of probable cloacal origin. The remaining 12 were also isolated from control samples and so were assumed to be contaminants (Additional file 1).

A virtually perfect correlation existed between the size of ARISA peaks and clone length (r = 1.000, F_1,95_ = 299,083, P < 0.001), although some discrepancies still existed (Table 1). Clone lengths differed from their corresponding ARISA peak sizes by a mean of 1.0 ± 3.6 bp (range −6 to +15 bp).

### Bacterial taxonomy

The resolution of OTU identification depended on both the strength of the BLAST match and the ability to resolve their respective phylogenetic positions (Additional file 2). The mean maximum BLAST score for each OTU was 199 ± 25 (range = 90 – 243). In 64.5% of cases we assigned an OTU to a specific genus, to a family in 14.5% of cases, to a sub-order in 2.6% of cases, to an order in 13.2% of cases, to a class in 2.6% of cases and to a phylum in 2.6% of cases. The most common phylum represented by the cloacal assemblages was Firmicutes (50.0%), followed by Actinobacteria (37.5%) and Proteobacteria (12.5% consisting of 4.7% Alphaproteobacteria, 3.1% Betaproteobacteria and 4.7% Gammaproteobacteria). The most common genera found were *Corynebacterium* (10.9%) and *Lactobacillus* (6.6%). The majority of OTUs identified in the control samples belonged to the class Alphaproteobacteria (58.3%). In addition, we also identified bacteria from the classes Betaproteobacteria (16.7%) and Gammaproteobacteria (8.3%) and from the phylum Firmicutes (16.7%).

### Bacterial assemblages in adults and chicks

**Figure 1:**
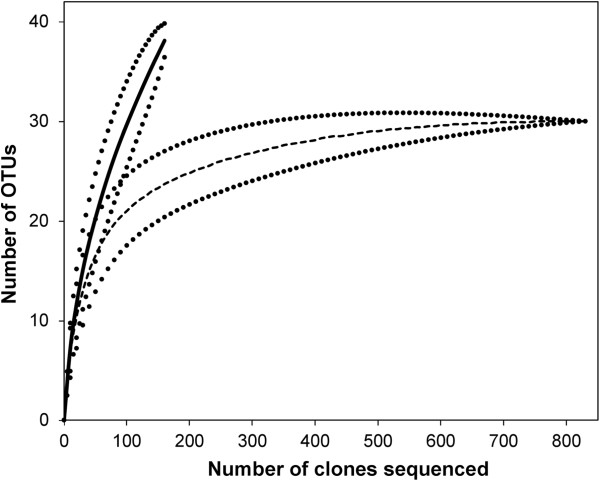
**Rarefaction curves of OTU richness and cloning effort of cloacal bacteria of adult and chicks.** Adults are represented by the dashed line and chicks by the solid line. Dotted lines represent 95% confidence limits.

**Figure 2:**
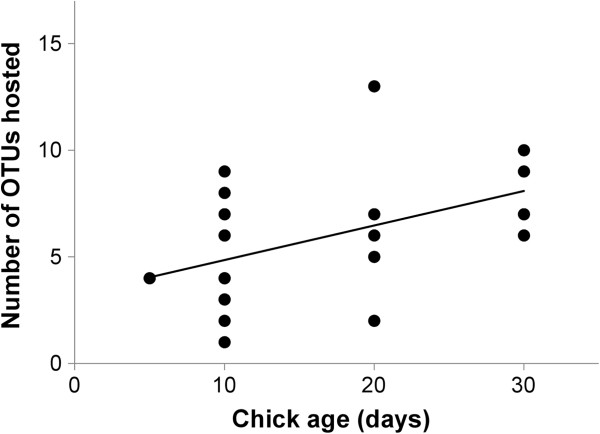
Age-related changes in the number of bacterial OTUs hosted by black-legged kittiwake chicks.

From our 23S rRNA cloning library, we identified 31 OTUs in adults and 40 OTUs in chicks, despite sequencing less than a quarter the number of chick to adult clones. Rarefaction analyses revealed that we had sampled the majority of common OTUs found in adults, but that many rare OTUs remained unidentified in chicks (Figure 1). Only seven OTUs were shared between the two age groups. Sixty three percent of OTUs identified in chicks were isolated from only single individuals while, in adults, 48% of OTUs were isolated from single individuals. From the ARISA data, chick age was positively correlated with number of OTUs hosted per individual (r = 0.452, F_1,19_ = 4.889, P = 0.039; Figure 2). In addition, adults hosted a higher number of OTUs per individual than chicks (mean number of OTUs per individual kittiwake: chicks = 5.8 ± 3.0 OTUs, adults = 9.2 ± 2.3 OTUs, F_1,43_ = 17.556, P < 0.001).

**Figure 3:**
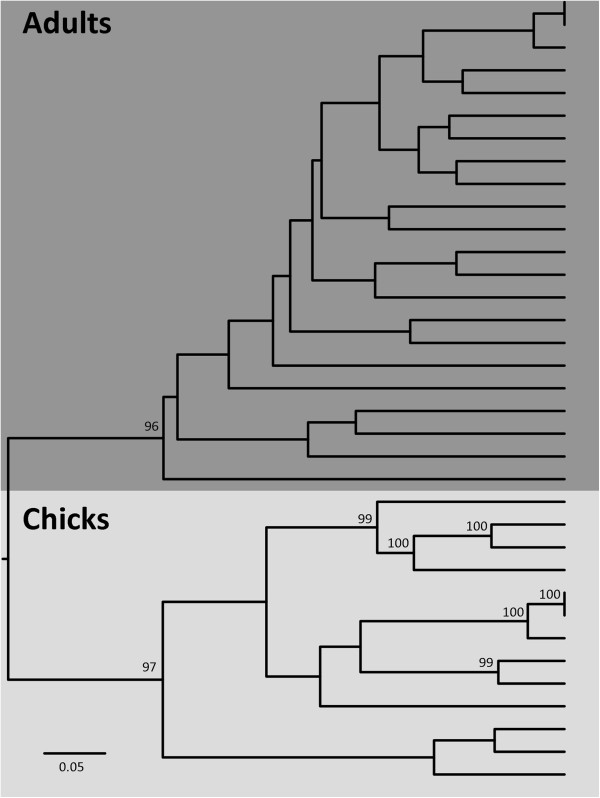
**Unifrac clustering analysis of bacterial assemblages in the cloacae of adult and chick black-legged kittiwakes.** Adults are represented by the dark grey box and chicks by the light grey box. Each leaf represents the bacterial assemblage of one individual kittiwake. Nodes with numbers were recovered more than 95% of the time during 100 permutations. Note that the analysis removed several chick individuals that harboured fewer than five OTUs (see Methods).

**Figure 4:**
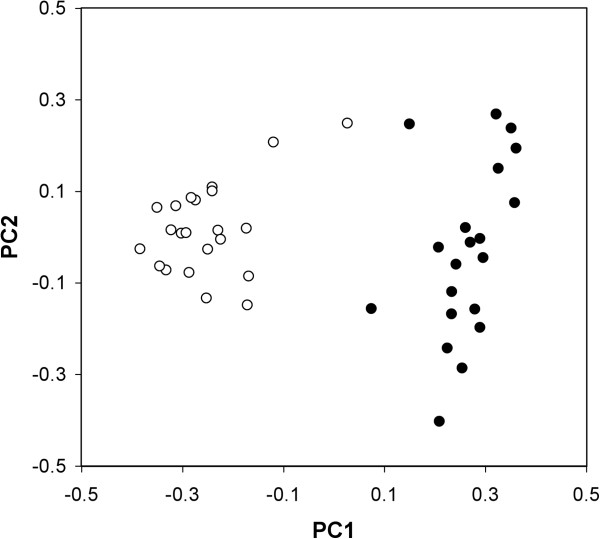
**First two principal coordinates extracted from a principal coordinate analysis of kittiwake cloacal bacterial assemblages.** Chicks are represented by closed circles and adults by open circles. The first principal component explained 45.6% of the variation, while the second explained 12.0%.

Analyses in Unifrac revealed strong clustering of the sequences by age (Figure 3). In addition, principal coordinates analysis separated chicks and adults along the first axis explaining 45.6% of the variation (PC1 scores – chicks: 0.264 ± 0.070, adults: -0.252 ± 0.091, F_1,41_ = 430.5, P < 0.001; Figure 4), but not along the second axis, which explained 12.0% of variation, or the third axis, explaining 7.9% of variation (PC2 scores – chicks: -0.019 ± 0.195, adults: 0.018 ± 0.099, Z = −0.875, P = 0.382; PC2 scores – chicks: -0.019 ± 0.195, adults: 0.018 ± 0.099, Z = −0.462, P = 0.664).

The clustering by age was statistically significant (P test in UniFrac: P = 0.007), although the clusters did not represent evolutionary divergence of bacteria between the age classes (Unifrac significance: P = 0.104). Lineage-specific analysis suggested that the age differences in bacterial assemblages were due to the presence of several OTUs in only one age class. For example, various OTUs of the genus *Corynebacterium* were abundant in adults (six OTUs identified, many of which were present in most individuals), but virtually absent in chicks (one OTU identified in only one individual; P < 0.001). Similarly, certain OTUs were only found in adults (e.g. OTU 1: *Peptoniphilus* sp., P < 0.001; OTU 18: Actinomycetaceae sp., P < 0.001; OTU 46: Clostridiales sp., P < 0.001), while others were unique to or more abundant in chicks (e.g. OTU 27 and 32; *Bacillus* sp., P < 0.001; OTU 37: *Escherichia* sp., P < 0.001).

## Discussion

We identified 64 bacterial OTUs in the cloacae of adult and chick black-legged kittiwakes, a majority of which were identified as Firmicutes and Actinobacteria, while a smaller proportion belonged to the Proteobacteria. A greater number of total OTUs were identified in chick cloacae than in adults. However, the number of OTUs hosted per individual increased with age. Older chicks hosted more OTUs per individual than younger chicks and adults hosted more OTUs per individual than chicks. Surprisingly very little overlap existed in the bacterial assemblages between chicks and adults. Only seven of 64 OTUs were shared between the two age groups (e.g. OTU 3: order Lactobacillales, OTU 9: genus: *Lactobacillus* and OTU 37: genus *Escherichia*), while the vast majority were found exclusively in only chicks or adults. For example, *Corynebacterium* was the most common genus of bacteria identified in adults, but it was virtually absent from chicks. This pronounced difference between adults and chicks resulted in strong statistical clustering of bacterial assemblages according to host age.

ARISA has been widely used to provide important insights in a wide range of fields within microbiology e.g. [34-38]. Coupled with a clone library for species identification, it represents a highly relevant research tool to allow the rapid and inexpensive characterisation of environmental bacterial assemblages. Despite the advantages of ARISA, this technique has some limitations (which are not all necessarily restricted to ARISA). For example, biases inherent during DNA extraction and PCR are also known to affect the apparent composition of bacterial assemblages [39,40]. ARISA can potentially underestimate species richness, as eight percent of bacterial species are known not to have the 23S and 16S rRNA organised in an operon (i.e. they have no intergenic spacer region) or have very large IGS lengths that cannot be detected by ARISA [29]. In addition, divergent bacteria may share the same IGS length and therefore be associated with the same ARISA peak [this study, [29]. In contrast, some species have several operons in their genomes resulting in multiple ARISA peaks for single species and potentially leading to overestimates in diversity [29]. In our dataset, we conservatively assumed that any two clones with the same 23S sequence derived from the same OTU. We consequently identified many OTUs with multiple operons (1–9 operons were identified per OTU). We also only included OTUs in our analyses for which we could confidently assign ARISA peaks, resulting in several genuine OTUs being excluded. This conservative approach means that our estimates of OTU richness are likely to represent a minimum. However, these biases apply equally for both chicks and adults and we were still able to identify a relatively large number of OTUs suitable for community-level comparisons.

The establishment of bacterial communities in the gastrointestinal tract of young animals is characterised by a high turnover of many transient species and large changes in community structure over short periods of time. For example, González-Braojos et al. 2012 [23] found that, in faecal sacs of nestling pied flycatchers (*Ficedula hypoleuca*), Enterobacteriaceae loads decreased when the nestlings aged from 7 to 13 days, while Enterococci loads concurrently increased. Age-related changes in the composition of other important gastrointestinal microbes, such as fungi, are also known to occur e.g. [25]. The rapid changes in bacterial community structure in young animals may arise due to a number of reasons including resource competition between bacterial species, shifts in host diet or age-related variation in the chemical and physiological state of the gastrointestinal tract [8,10,23]. For example, in humans, the early colonisation of the gut by facultative anaerobes (e.g. Enterococci and Enterobacteria) reduces gut oxygen levels which allows anaerobic bacteria to become established [8]. Eventually, gastrointestinal bacterial communities are known to transition to a stable adult state [7]. Adult bacterial communities may differ from those of young individuals due to the more developed immune system of adults [41-43], the low mobility of young animals resulting in a restricted environment from which to obtain bacteria or contrasting chemical and anatomical cloacal environments that are differentially hospitable or hostile to various bacteria [7,8].

Our data support these findings. For example, the fact that the chick rarefaction curve failed to plateau and that more OTUs were unique to individual chicks than adults suggests that chicks host more rare, and presumably transient, bacterial species than adults. Second, our finding that adults host a greater number of OTUs per individual than chicks, and that the number of OTUs hosted by chicks increases with age, is in accordance with previous studies that have shown that species richness in bacterial assemblages increases as animals reach adulthood e.g. [19,20,26]. Last, we identified substantial variation in bacterial assemblage composition between chicks and adults, highlighting the dynamic nature of bacterial communities within the gut. It is, unfortunately, not possible to deduce the fitness consequences on hosts of age-related changes in bacterial microbiota from our genetic data, especially given the great intrageneric diversity in ecological roles and pathogenicity of bacteria. It, for example, remains unknown why bacteria of the genus *Corynebacterium* are so prevalent in adults, but almost absent in chicks. However, some inferences can still be made. The seven OTUs shared between chicks and adults may be beneficial or commensal and therefore retained in the gastrointestinal microbiota as the hosts age. For example, two of the shared OTUs were Lactobacillales species, many of which are known to competitively exclude pathogenic bacteria and increase antibody levels, thus increasing immunity to pathogens [44,45]. Similarly, another shared OTU belonged to the genus *Escherichia*. *Escherichia* bacteria are common commensals in the gastrointestinal tract [46], which are known to rapidly colonise the gut of young birds [18,19,24].

The data generated in this study will provide new opportunities to investigate the causes and consequences of variation in bacterial assemblages in a wild bird species. Although much is known in domesticated bird species, relatively little is known about the acquisition of gastrointestinal bacteria assemblages in wild birds. For example, assemblages are known to vary with both external factors, such as nutrition e.g. [47] and environment e.g. [48], and host-related traits such as genotype e.g. [49], body condition e.g. [48], immune system e.g. [50], and sex and mating behaviour e.g. [13]. Our data will allow us to build on these studies with large experimental data sets to explore how specific bacterial species are acquired (e.g. via cross-fostering experiments to ascertain how environment and genotype affect microbial assemblages in chicks) and how they impact on host condition and fitness (e.g. whether individuals that host different bacterial assemblages cf. enterotypes: [51] differ in condition or reproductive success).

## Conclusions

The striking difference in bacterial assemblages between chicks and adults suggests that despite sharing the same nesting environment and being fed regurgitated food by their parents, others factors affect the acquisition of cloacal bacterial assemblages in kittiwakes. Although several previous studies in both domestic e.g. [17,19,20] and wild birds e.g. [10,24,25] have demonstrated age-differences in gastrointestinal bacteria, we have done so at the community level rather than the single-species level. This approach has highlighted how strongly assemblages can differ between age-classes, which was not as apparent in previous studies using only culture-based methods that target a limited number of bacterial species but see [10]. As gastrointestinal bacteria have important functions in digestion, immune functions and general health, the characterisation of the acquisition of bacteria comprises a crucial component in achieving a more comprehensive understanding of the causes and consequences of variation in bacterial communities in wild animals.

## Abbreviations

ARISA: Automated ribosomal intergenic spacer analysis; BLAST: Basic local alignment search tool; DNA: Deoxyribonucleic acid; dNTPs: Deoxynucleotide triphosphates; IGS: Intergenic spacer; MgCl2: Magnesium chloride; OTU: Operational taxonomic unit; PCR: Polymerase chain reaction; rRNA: ribosomal ribonucleic acid; SD: Standard deviation

## Competing interests

The authors declare that they have no competing interests.

## Authors’ contributions

The study was designed by JW, RW and ED and fieldwork was performed by JW, TM, SL, PB and SH. HB conducted the laboratory work. YM provided advice for the genetic analyses and WvD and HB analysed the data. WvD wrote the manuscript. All authors contributed to and approved the final manuscript.

## Supplementary Material

Additional file 1 Identity of OTUs isolated from control samples that are assumed to be contaminants. Click here for file

Additional file 2 23S rRNA phylogenetic trees for the 76 identified bacterial OTUs isolated from black-legged kittiwake cloacae. Click here for file
