# Supplementary material for: Age-related differences in the cloacal microbiota of a wild bird species
Source: BMC Ecol. 2013 Mar 25;13:11. doi: 10.1186/1472-6785-13-11 (PMC3668179; doi:10.1186/1472-6785-13-11)
Supplement: Additional file 1 — Identity of OTUs isolated from control samples that are assumed to be contaminants. [file 1472-6785-13-11-S1.doc]

**Additional file 1. Identity of OTUs isolated from control samples that are assumed to be contaminants.** For each OTU, the following variables are displayed: OTU identification number (following on from the OTU numbers outlined in Table 1), ARISA peak size, clone fragment length, the difference between the latter two variables, the species identity of the most similar sequence in GenBank as determined by the BLAST algorithm, the maximum BLAST score, the most derived phylogenetic position of the OTU and the phylum to which the OTU belongs. In some cases, we could not determine which clone represented a particular ARISA peak (indicated by a blank space in the “ARISA OTU” column).

| **OTU** | **ARISA**  **peak** | **Clone** | **Difference** | **BLAST match** | **BLAST score** | **Most derived phylogenetic position** | **Phylum** |
| --- | --- | --- | --- | --- | --- | --- | --- |
| 65 | ? | 333 |  | *Bacillus pumilus* | 213 | Genus: *Bacillus* | Firmicutes |
| 66 | 542 | 543 | +1 | *Carnobacterium* sp. | 215 | Genus: *Carnobacterium* | Firmicutes |
| 67 | 677 | 677 | 0 | *Stenotrophomonas maltophilia* | 213 | Genus: *Stenotrophomonas* | Proteobacteria |
| 67 | 678 | 678 | 0 | *Stenotrophomonas maltophilia* | 213 | Genus: *Stenotrophomonas* | Proteobacteria |
| 68 | 722 | 724 | +2 | *Variovorax paradoxus* | 217 | Family: Comamonadaceae | Proteobacteria |
| 69 | ? | 786 |  | *Bosea* sp. | 215 | Genus: *Bosea* | Proteobacteria |
| 69 | 825 | 825 | 0 | *Bosea* sp. | 215 | Genus: *Bosea* | Proteobacteria |
| 69 |  | 872 |  | *Bosea* sp. | 215 | Genus: *Bosea* | Proteobacteria |
| 70 | ? | 796 |  | *Brevundimonas subvibrioides* | 207 | Genus: *Brevundimonas* | Proteobacteria |
| 70 | ? | 800 |  | *Brevundimonas subvibrioides* | 207 | Genus: *Brevundimonas* | Proteobacteria |
| 71 | 799 | 802 | +3 | *Bosea* sp. | 202 | Genus: *Bosea* | Proteobacteria |
| 72 | ? | 802 |  | *Sphingopyxis alaskensis* | 204 | Family: Sphingomonadaceae | Proteobacteria |
| 73 | 819 | 824 | +5 | *Bordetella petrii* | 195 | Family: Alcaligenaceae | Proteobacteria |
| 74 | 860 | 860 | 0 | *Bradyrhizobium* sp. | 171 | Order: Rhizobiales | Proteobacteria |
| 74 |  | 908 |  | *Bradyrhizobium* sp. | 171 | Order: Rhizobiales | Proteobacteria |
| 75 | 888 | 901 | +13 | *Sphingomonas* sp. | 215 | Genus: *Sphingomonas* | Proteobacteria |
| 75 | 889 | 902 | +13 | *Sphingomonas* sp. | 215 | Genus: *Sphingomonas* | Proteobacteria |
| 75 | 890 | 903 | +13 | *Sphingomonas* sp. | 215 | Genus: *Sphingomonas* | Proteobacteria |
| 76 | 986 | 987 | +1 | *Pelagibacterium halotolerans* | 152 | Class: Alphaproteobacteria | Proteobacteria |
