# Supplementary material for: Age-related differences in the cloacal microbiota of a wild bird species
Source: BMC Ecol. 2013 Mar 25;13:11. doi: 10.1186/1472-6785-13-11 (PMC3668179; doi:10.1186/1472-6785-13-11)
Supplement: Additional file 2 — 23S rRNA phylogenetic trees for the 76 identified bacterial OTUs isolated from black-legged kittiwake cloacae. [file 1472-6785-13-11-S2.doc]

**Additional file 2. 23S rRNA phylogenetic trees for the 76 identified bacterial OTUs isolated from black-legged kittiwake cloacae.** a) Root tree, displaying the relationships between the phyla represented by the OTUs, b) phylum Actinobacteria, c) order Clostridiales (phylum Firmicutes), d) order Lactobacillales (phylum Firmicutes), e) class Alphaproteobacteria (phylum Proteobacteria), f) class Betaproteobacteria (phylum Proteobacteria) and g) class Gammaproteobacteria (phylum Proteobacteria). OTUs identified in adults, chicks or in the control samples are indicated by “Ad”, “Ch” and “Co”, respectively. Phylogenies were inferred using Maximum Likelihood and Bayesian methods. Thick-lined nodes represent those with high Maximum Likelihood bootstrap support (≥70%) and asterisks highlight nodes with Bayesian posterior probabilities ≥95%. The identity of each species is given in Table 1. Trees were rooted with *Fervidicoccus fontis* and *Thermotoga neapolitana*.

**
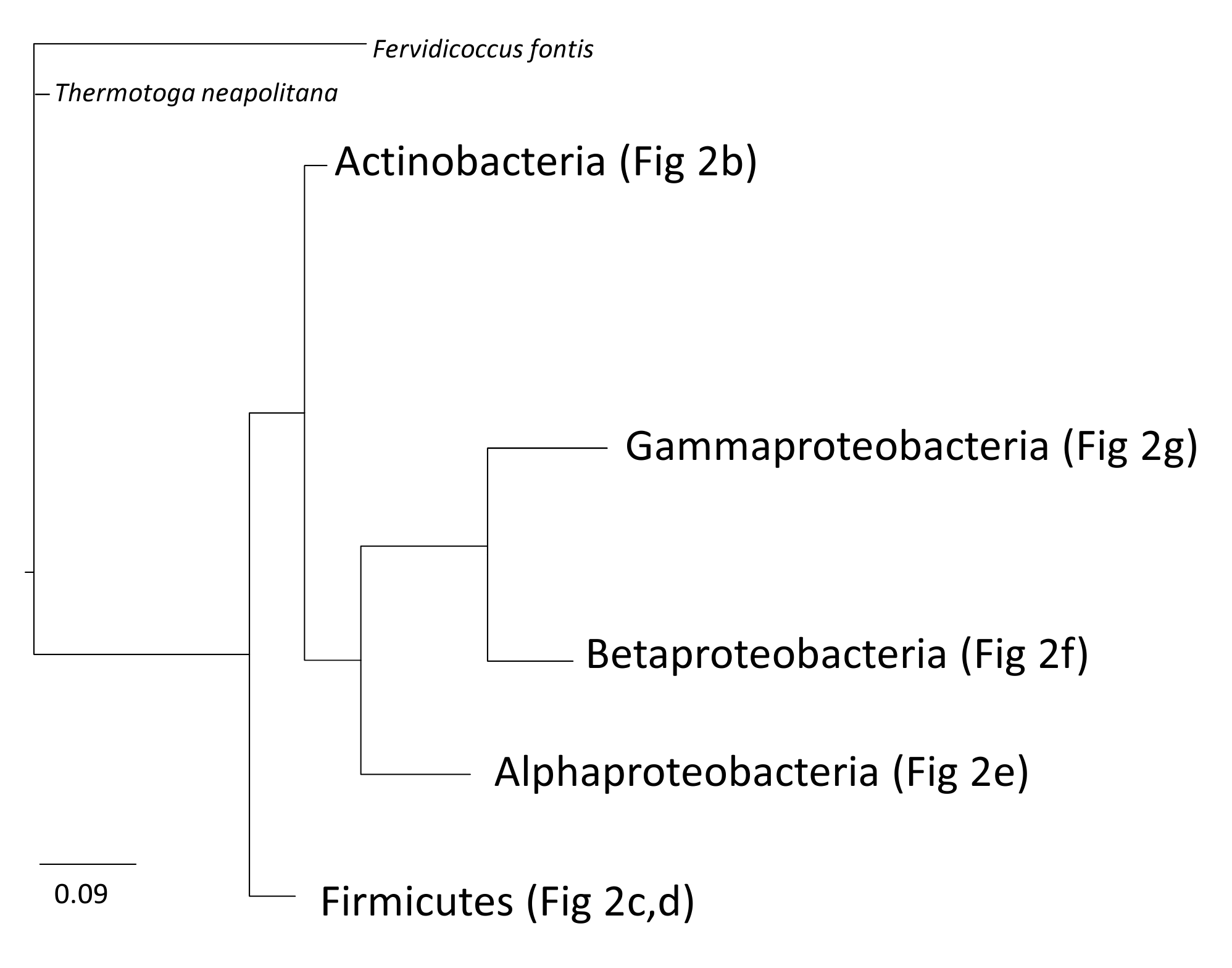
**

**Additional file 2a.**

**
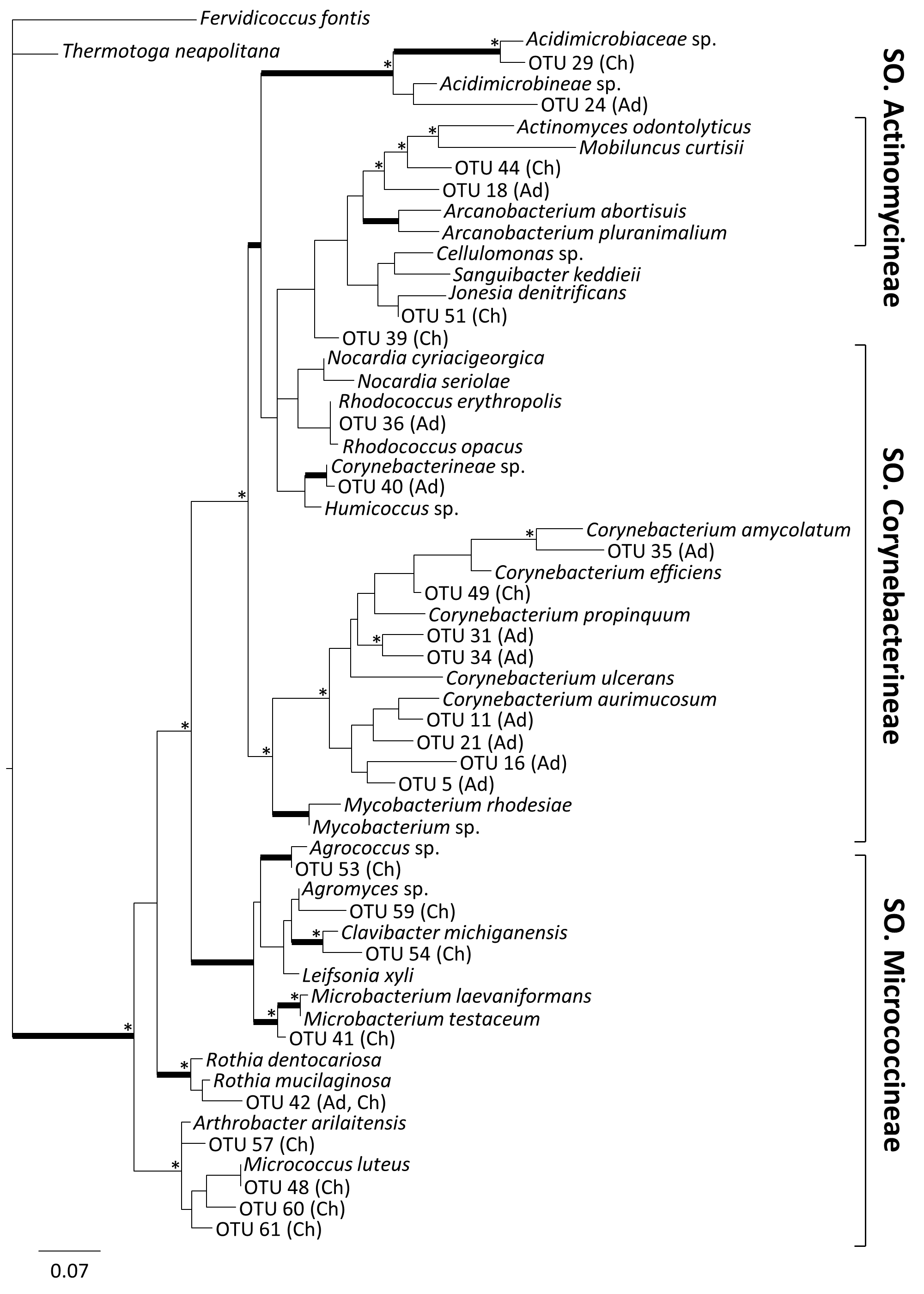
**

**Additional file 2b.**

**
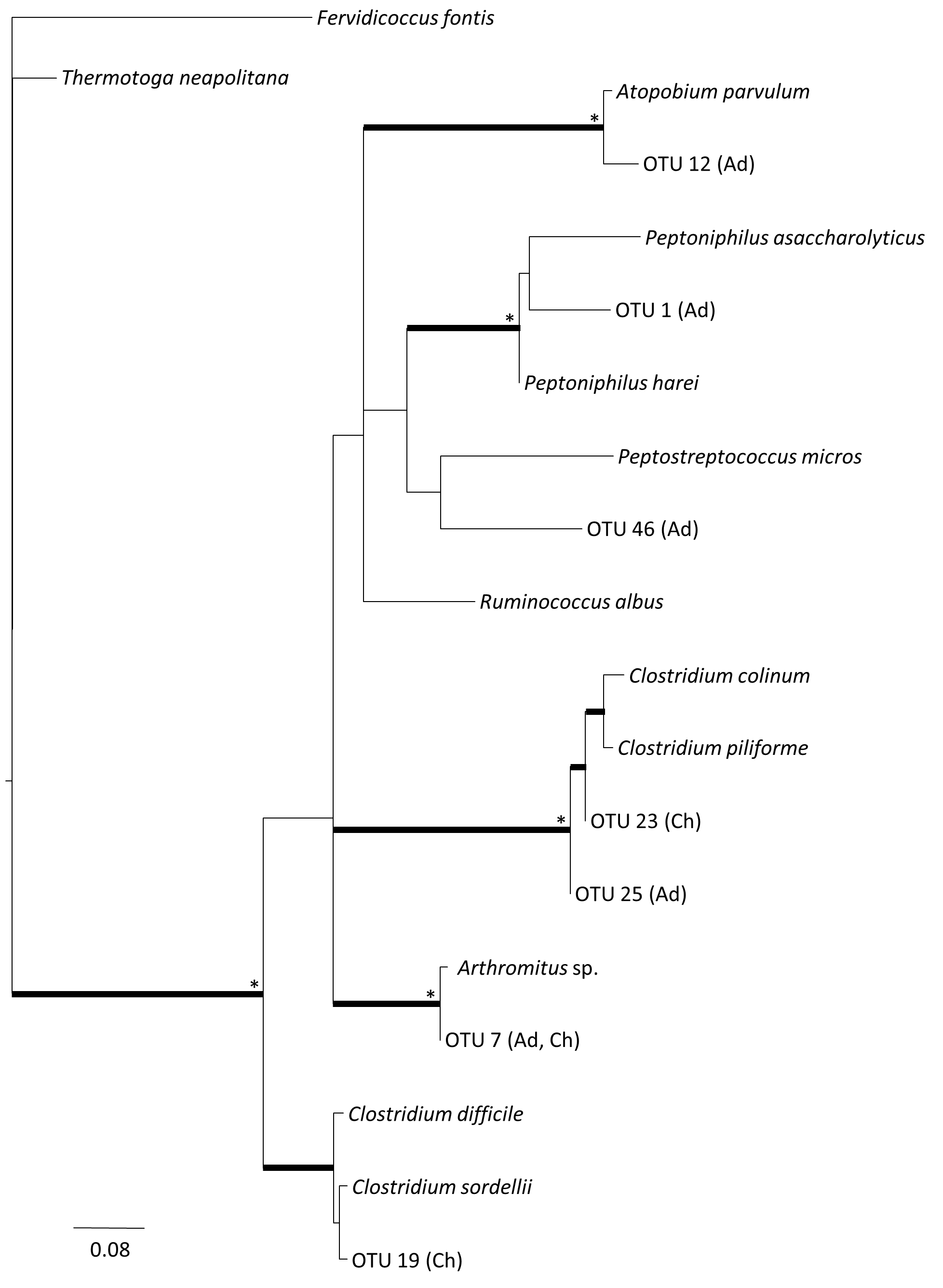
**

**Additional file 2c.**

**
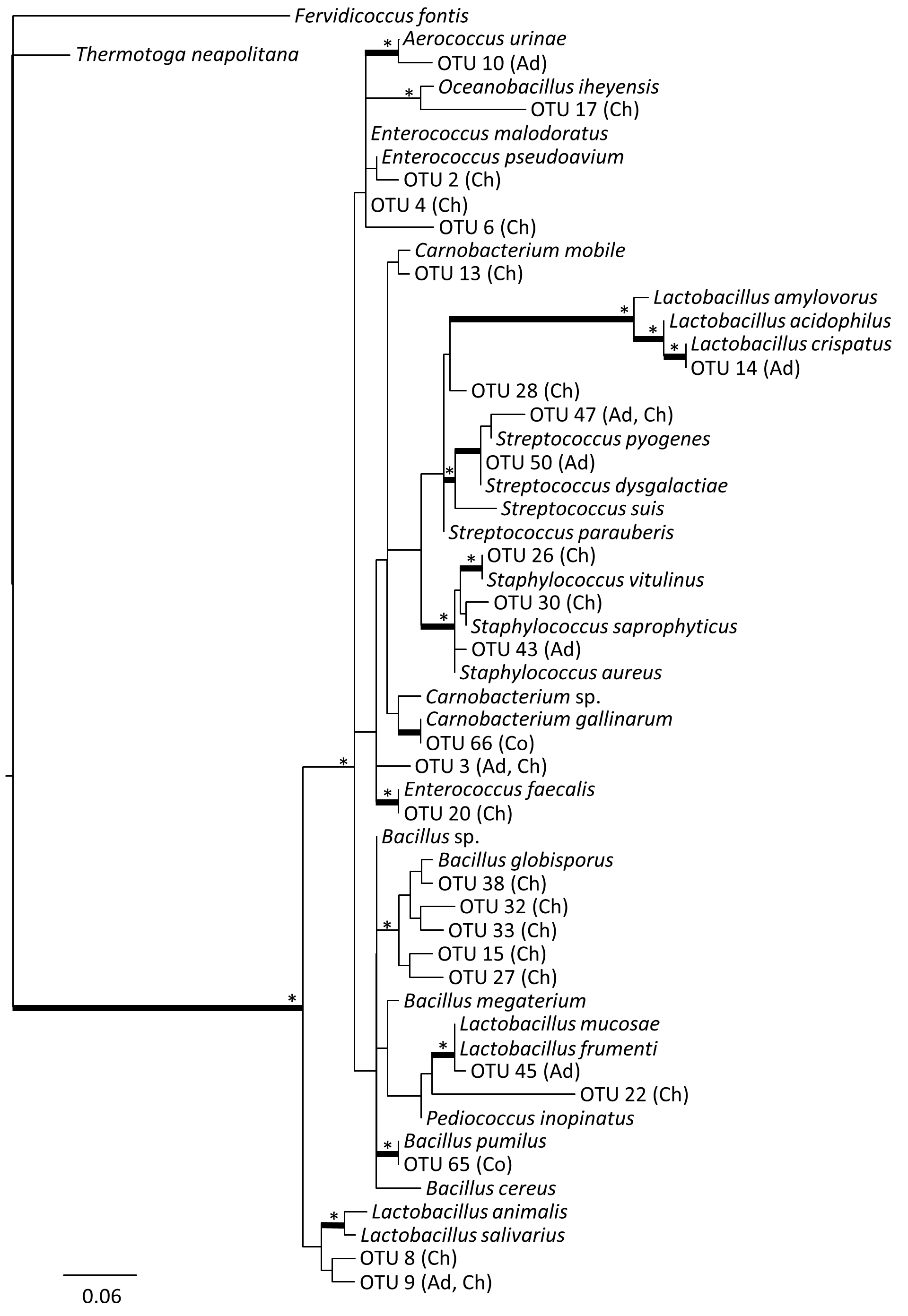
 Additional file 2d.**

**
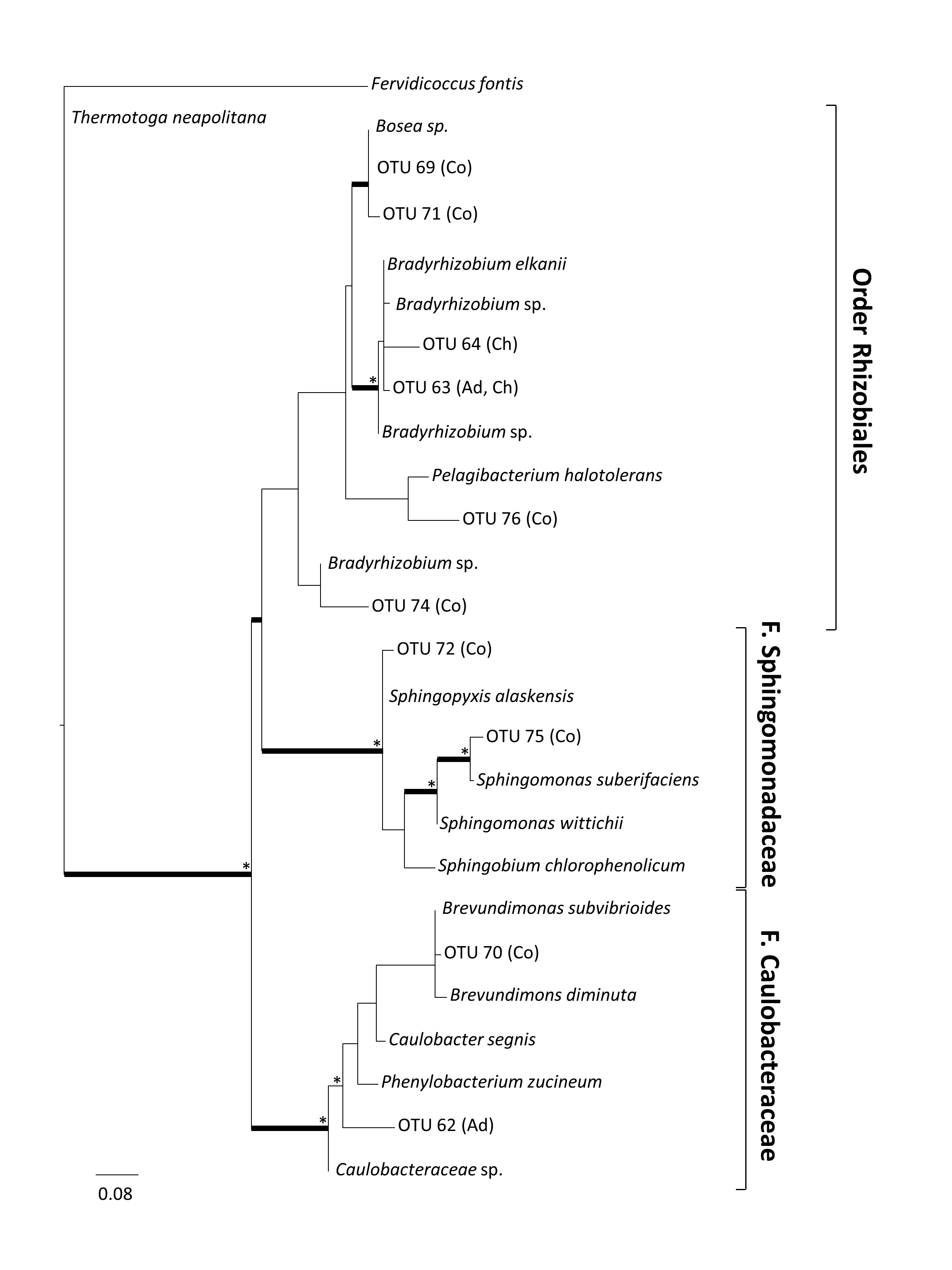
**

**Additional file 2e.**

**
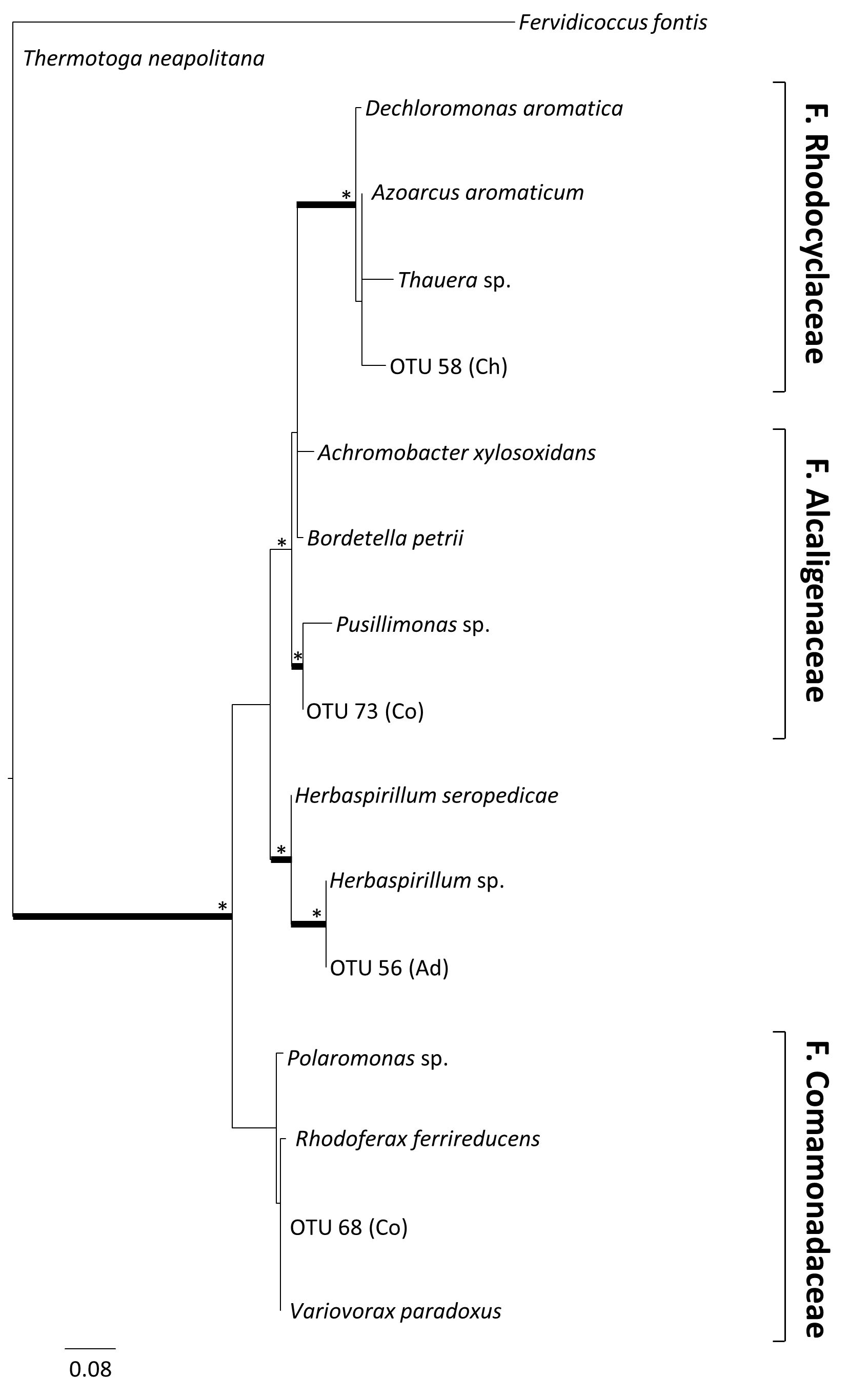
**

**Additional file 2f.**

**
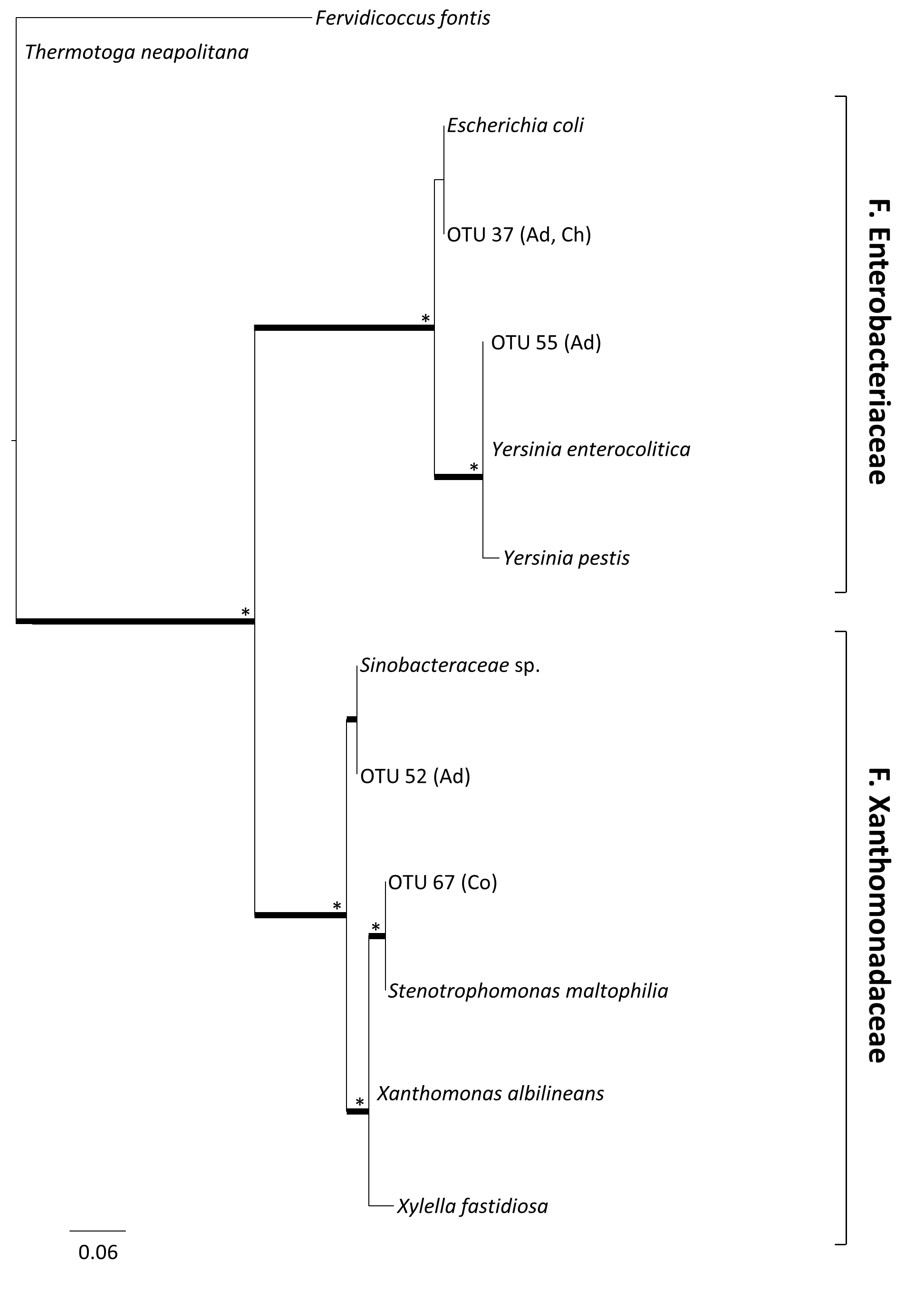
**

**Additional file 2g.**
